# Deterministic modelling of asymptomatic spread and disease stage progression in vaccine preventable infectious diseases

**DOI:** 10.1002/qub2.50

**Published:** 2024-07-14

**Authors:** Gabor Kiss, Salissou Moutari, Cara Mctaggart, Lynsey Patterson, Frank Kee, Felicity Lamrock

**Affiliations:** ^1^ Mathematical Sciences Research Centre School of Mathematics & Physics Queen’s University Belfast Belfast UK; ^2^ Public Health Agency Belfast UK; ^3^ Centre for Public Health Queen’s University Belfast Belfast UK

## Abstract

This study introduces a deterministic formulation for modelling the asymptotic spread of a vaccine preventable disease as well as the different stages for the progression of the disease. We derive the formula for the associated basic reproduction number. To illustrate the proposed model, we use data from the 2017–2018 diphtheria outbreak in Yemen and fit the parameters of the model. A sensitivity analysis of the basic reproduction number, with respect to the model parameters, show that this number increases with an increase of the transmission rate while this number decreases when vaccination rate increases.

## 1 INTRODUCTION

Infectious diseases have affected humankind in many different ways, either through diseases spreading within livestock (bovine tuberculosis), from animal hosts to humans (malaria), ingestion of contaminated food or water (typhoid fever) or through direct human to human interactions (influenza). Mathematical epidemiology has been playing an important role in understanding and controlling the spread of infectious diseases since the work of Daniel Bernoulli in 1760 [1, 2, 3]. Since then, in particular from the beginning the 20th century, various deterministic and non‐deterministic approaches have been developed to understand how infectious disease spread. These models have been used to mitigate the devastating effects of many infectious disease outbreaks, providing policymakers with the tools to plan and deliver effective control or vaccination strategies [4, 5, 6, 7].

One of the most widely used deterministic models, based on the 1927 work of Kermack and McKendrick [8], is the so‐called susceptible‐infectious‐removed (SIR) model.^1^ The SIR partition of a population has been used widely to model the dynamics of many infectious diseases. Although the model is relatively simple, it is still a powerful tool to understand the population level behaviour of a novel communicable disease when very little is known about it. However, usually, over time, an increased understanding of the in‐host disease progression can serve as the basis to derive more detailed models by considering various additional compartments and the transition processes between them to reflect the stages where a person can potentially spend some time while progressing from susceptible to other significant stages of the infection. Henceforth, faithful mathematical models for the spread of the disease, at the population level, can be built, analysed and utilised to control infectious disease epidemics. For instance, in the case of COVID‐19, once the presence of latent period or asymptomatic disease spread and uncertainty in testing had been evidenced, these aspects were incorporated into various mathematical models [9, 10].

One of the most important advantages of mathematical epidemiology of infectious diseases is that it is not the disease or the pathogen itself which is considered during the model building process but rather more general features such as within‐population contact rates, recovery rates as well as other transmission or transfer rates between compartments. In addition, once a model is formulated, mathematical techniques can be used to derive, in terms of model parameters, the so‐called basic reproduction number, denoted by **R**_0_, which is the number of generated secondary infections. The number **R**_0_, which has threshold value of 1, is an indicator of whether an emerging communicable disease can spread in the considered population. If the considered features of a model are generic enough, it can be used to model and control the outbreak of various diseases regardless of whether the infectious agent is a virus, bacterium, fungus, protozoan or helminth.

In this study, we consider a model based on ordinary differential equations, which has the potential of capturing the dynamics of various vaccine preventable infections at (not necessarily human) population level. As usual, in deterministic modelling of epidemiological processes, the population is assumed to be large enough for this purpose. Furthermore, since many infectious diseases feature a so‐called latent period, that is, the time between infection and the onset of symptoms, we consider an exposed compartment and we assume that there is no difference between the latent and the incubation period. An infected individual becomes infectious as soon as at least one of the possible symptoms is present. We also incorporate the possibility of asymptomatic infection; therefore, the time from being infected to becoming infectious is referred as incubation time in this paper. For the symptomatic cases, we assume the possibilities of mild and severe cases. As a final stage of the disease progression, a ‘removed’ compartment is considered. We assume that a life‐long immunity to the pathogen is developed after an exposure to it.

The paper is organised as follows. In Section 2, we present a detailed derivation of the model and derive the formula for the basic reproduction number. In Section 3, we fit our model parameters to the data of the 2017–2018 diphtheria outbreak in Yemen. Furthermore, we perform a sensitivity analysis of the basic reproduction number in terms of the parameters of the models. In Section 4, we summarise our findings and briefly discuss some possible extensions of the proposed model.

## 2 RESULTS

To build a model, which reflects the assumptions outlined in the Introduction, we consider a population of size *N* > 0, which consists of the union of seven disjoint sub‐populations as follows. First, we assume that individuals can leave any of the compartments with a rate of *d* > 0, representing the natural death (we do not consider disease induced mortality in this paper). The class of susceptible, *S*, is one of the groups of individuals who can be infected. We assume that new susceptibles enter the population via birth. And at any given time, the number of newborns is Λ*N*, Λ > 0, where Λ is the birth rate. However, we only introduce birth and death so that the assumptions in ref. [11] are satisfied when formulating **R**_0_ related statements. We also assume that susceptibles are vaccinated at a rate of *ν* > 0, and the compartment of the vaccinated, *V*, comprises the other group of individuals who can get the infection although the rate of transmission, *β* > 0, in this group is reduced by a factor *σ* compared to the group *S*. The loss of immunity, represented by *σ*, stems from an imperfect vaccine^2^ or the waning of the immunity provided by the vaccine.

**FIGURE 1:**
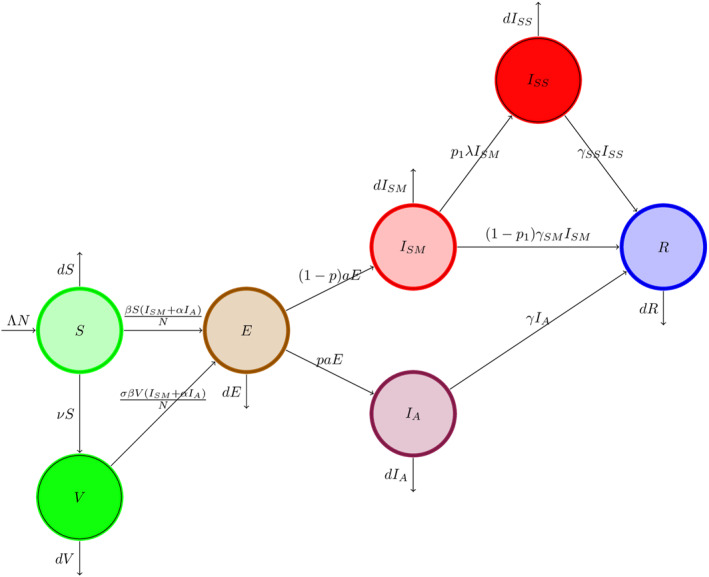
Transmission diagram.

In our model, the average incubation time is $\frac{1}{a},\;a>0$, that is, the exposed individuals are moving from compartment *E* into one of the groups transmitting the disease at a rate *a*. In the next stage of the disease progression, an exposed individual can become infectious without symptoms with a probability *p* ∈ [0, 1]. These individuals spend $\frac{1}{\gamma },\;\gamma >0$, units of time in compartment *I*_*A*_ and the recovery rate from this compartment is *γ*. We assume that the infectiousness of individuals in this class is reduced by a factor *α* ∈ [0, 1], compared to the other infectious compartment. The other branch in disease progression, receives exposed individuals from class *E* into compartment *I*_*SM*_, representing the mild symptomatic cases, with a probability 1 − *p*. In this group, individuals are assumed to recover with probability 1 − *p*_1_, *p*_1_ ∈ [0, 1], and with a rate of *γ*_*SM*_, *γ*_*SM*_ > 0; that is, the average recovery time in this class is $\frac{1}{\gamma_{SM}}$ units of time. However, we assume that after $\frac{1}{\lambda }$ units of time, mild cases develop into severe ones, with probability *p*_1_. That is, the transfer rate from *I*_*SM*_ into *I*_*SS*_, the compartment representing the severe cases, is *λ*. For individuals in *I*_*SS*_, we also assume that they do not contribute to the disease transmission as a result of voluntary or disease‐spread mitigating interventions induced by, for instance, removing social interactions. Individuals are moving from compartment *I*_*SS*_ into compartment *R* with a rate of *γ*_*SS*_ > 0, that is, on average, an individual spends $\frac{1}{\gamma_{SS}}$ units of time in *I*_*SS*_.^3^ Finally, we assume that the natural immunity provides lifelong protection against the disease in question, that is, the class of recovered, *R*, is the terminal compartment of the disease progression. These assumptions are visualised in Figure 1.

Based on our assumptions, we formulate the following model of differential equations:

(1) $$\begin{array}{cc} \dot{S} & =\Lambda N-\frac{\beta S\left(I_{SM}+\alpha I_{A}\right)}{N}-\nu S-dS\\ \dot{V} & =\nu S-\frac{\sigma \beta V\left(I_{SM}+\alpha I_{A}\right)}{N}-dV\\ \dot{E} & =\frac{\beta S\left(I_{SM}+\alpha I_{A}\right)}{N}+\frac{\sigma \beta V\left(I_{SM}+\alpha I_{A}\right)}{N}-aE-dE\\ {\dot{I}}_{A} & =paE-\gamma I_{A}-dI_{A}\\ {\dot{I}}_{SM} & =\left(1-p\right)aE-\left(1-p_{1}\right)\gamma_{SM}I_{SM}-p_{1}\lambda I_{SM}-dI_{SM}\\ {\dot{I}}_{SS} & =p_{1}\lambda I_{SM}-\gamma_{SS}I_{SS}-dI_{SS}\\ \dot{R} & =\gamma I_{A}+\left(1-p_{1}\right)\gamma_{SM}I_{SM}+\gamma_{SS}I_{SS}-dR, \end{array}$$

where, $\Lambda ,\beta ,\nu ,\sigma ,\alpha ,a,p,p_{1},\lambda ,\gamma ,d\in R^{+}$, and $N\in R^{+}$ is the size of the population. The meaning of the variables and the parameters, in Equation (1), is summarised in Tables 1 and 2, respectively. Once an initial condition, $\left(S_{0},V_{0},E_{0},{I_{A}}_{0},{I_{SM}}_{0},{I_{SS}}_{0},R_{0}\right)$, is specified, (1) has a unique solution, see ref. [16]. In the rest of this paper, we assume that,

(2) Λ=d,

which implies that the total population,

$$N\left(t\right)=S\left(t\right)+V\left(t\right)+E\left(t\right)+I_{A}\left(t\right)+I_{SM}\left(t\right)+I_{SS}\left(t\right)+R\left(t\right),$$

is constant. This statement can be easily verified by taking the sums of both sides of the equations in (1). Similarly, it can be easily shown that while Λ > *d* implies an exponentially growing, Λ < *d* implies an exponentially decaying total population. In what follows, $f:R_{+}^{7}\to R_{+}^{7}$ denotes the right hand side of (1).

**TABLE 1:** State variables of Equation (1).

Variable	Meaning
*S*(*t*)	The number of susceptible individuals at time *t* in the population
*V*(*t*)	The number of vaccinated individuals at time *t*
*E*(*t*)	The number of exposed individuals at time *t*
*I*_*A*_(*t*)	The number of asymptomatically infective individuals at time *t*
*I*_*SM*_(*t*)	The number of infective individuals with mild symptoms at time *t*
*I*_*SS*_(*t*)	The number of infected individuals with severe symptoms at time *t*
*R*(*t*)	The number of recovered individuals at time *t*

**TABLE 2:** Parameters in Equation (1) and their ranges used in Section 3.3.

Parameter	Description	Range	Refs
*β*	Transmission rate (d^−1^)	0.01–1	‐
*ν*	Vaccination rate (d^−1^)	$\frac{0.3}{365}-\frac{0.8}{365}$	[12]
*σ*	Transmission rate reduction in vaccinated	0.01–0.1	[13]
*α*	Infectiousness reduction in asymptomatic	0.59–0.87	[13]
*a* ^−1^	Average incubation period (d)	2–5	[14]
*p*	Probability of being asymptomatic	0.83–1	‐
*p*_1_	Probability of developing severe symptoms	0.1–0.25	[13]
*λ* ^−1^	Time to develop severe symptoms (d)	7–14	[13]
*γ* ^−1^	Asymptomatic infectious period (d)	17–20	[13]
$\gamma_{SM}^{-1}$	Mild symptomatic infectious period (d)	5–14	‐
$\gamma_{SS}^{-1}$	Severe symptomatic infectious period (d)	5–14	‐
Λ	Birth rate (d^−1^)	$\frac{0.031275}{365}$	[15]
*d*	Natural death rate (d^−1^)	$\frac{0.031275}{365}$	‐

### 2.1 The basic reproduction number

In order to find the formula of the basic reproduction number of (1), we use the method described in ref. [11]. A more direct approach to find **R**_0_, together with the biological interpretations of some of the matrices used in the calculations below, can be found in ref. [17].

First, let us reorder the compartments as follows: (*E*, *I*_*A*_, *I*_*SM*_, *I*_*SS*_, *S*, *V*, *R*). The method applied below is based on the so‐called disease free equilibrium (DFE), which is the solution of the following algebraic equation:

$$\begin{array}{cc} 0 & =\frac{\beta S\left(I_{SM}+\alpha I_{A}\right)}{N}+\frac{\sigma \beta V\left(I_{SM}+\alpha I_{A}\right)}{N}-aE-dE\\ 0 & =paE-\gamma I_{A}-dI_{A}\\ 0 & =\left(1-p\right)aE-\left(1-p_{1}\right)\gamma_{SM}I_{SM}-p_{1}\lambda I_{SM}-dI_{SM}\\ 0 & =p_{1}\lambda I_{SM}-\gamma_{SS}I_{SS}-dI_{SS}\\ 0 & =\Lambda N-\frac{\beta S\left(I_{SM}+\alpha I_{A}\right)}{N}-\nu S-dS\\ 0 & =\nu S-\frac{\sigma \beta V\left(I_{SM}+\alpha I_{A}\right)}{N}-dV\\ 0 & =\gamma I_{A}+\left(1-p_{1}\right)\gamma_{SM}I_{SM}+\gamma_{SS}I_{SS}-dR \end{array}$$

where it is assumed that *E* = *I*_*A*_ = *I*_*SM*_ = *I*_*SS*_ = *R* = 0, that is,

$$\text{DFE}=\left(0,0,0,0,\frac{\Lambda N}{d+\nu },\frac{\Lambda \nu N}{d\left(d+\nu \right)},0\right)$$

which, after using our assumption (2), simplifies as follows:

$$\text{DFE}=\left(0,0,0,0,\frac{dN}{d+\nu },\frac{\nu N}{d+\nu },0\right).$$

Therefore, at the DFE, *S* + *V* = *N*. Next, using the reordered compartments, we rewrite *f*, the right hand side of (1), as *f* = **F** − **V**, where

(3) $$F:R_{+}^{7}\to R_{+}^{7},\left(\begin{array}{c} E\\ I_{A}\\ I_{SM}\\ I_{SS}\\ S\\ V\\ R \end{array}\right)\mapsto \left(\begin{array}{c} \frac{\beta S\left(I_{SM}+\alpha I_{A}\right)}{N}+\frac{\sigma \beta V\left(I_{SM}+\alpha I_{A}\right)}{N}\\ 0\\ 0\\ 0\\ 0\\ 0\\ 0 \end{array}\right)$$

and

(4) $$V:R_{+}^{7}\to R_{+}^{7},\left(\begin{array}{c} E\\ I_{A}\\ I_{SM}\\ I_{SS}\\ S\\ V\\ R \end{array}\right)\mapsto \left(\begin{array}{c} aE+dE\\ \gamma I_{A}+dI_{A}\\ -(1-p)aE+\left(1-p_{1}\right)\gamma_{SM}I_{SM}+p_{1}\lambda I_{SM}+dI_{SM}\\ -p_{1}\lambda I_{SM}+\gamma_{SS}I_{SS}+dI_{SS}\\ -\Lambda N+\frac{\beta S\left(I_{SM}+\alpha I_{A}\right)}{N}+\nu S+dS\\ -\nu S+\frac{\sigma \beta V\left(I_{SM}+\alpha I_{A}\right)}{N}+dV\\ -\gamma I_{A}-\left(1-p_{1}\right)\gamma_{SM}I_{SM}-\gamma_{SS}I_{SS}+dR \end{array}\right).$$

It is relatively easy to see that *f*, **F** and **V** satisfy the conditions (A1)–(A5) in ref. [11]. In particular, when **F**(*x*) ≡ 0, the set of eigenvalues of *Df*(*DFE*),

$$\left\{-d,-a-d,-\gamma -d,-\gamma_{SS}-d,-d-\gamma_{SM}\left(1-p_{1}\right)-\lambda p_{1},-d-\nu \right\}$$

are all negative, as (A5) in ref. [11] assumes. Notice that −*d* is a zero of multiplicity two of the characteristic equation of *Df*(DFE). Therefore, by Lemma 1 in ref. [11], when the Jacobian matrices of **F** and **V** are calculated at the DFE, we have,

$$DF\left(\text{DFE}\right)=\left(\begin{array}{cc} F & 0\\ 0 & 0 \end{array}\right)\;\text{and}\;DV\left(\text{DFE}\right)=\left(\begin{array}{cc} V & 0\\ J_{3} & J_{4} \end{array}\right),$$

respectively, with

$$F=\left(\begin{array}{cccc} 0 & \frac{\alpha \beta \Lambda \nu \sigma }{d\left(d+\nu \right)}+\frac{\alpha \beta \Lambda }{d+\nu } & \frac{\beta \Lambda \nu \sigma }{d\left(d+\nu \right)}+\frac{\beta \Lambda }{d+\nu } & 0\\ 0 & 0 & 0 & 0\\ 0 & 0 & 0 & 0\\ 0 & 0 & 0 & 0 \end{array}\right),\;J_{4}=\left(\begin{array}{ccc} d+\nu & 0 & 0\\ -\nu & d & 0\\ 0 & 0 & d \end{array}\right)$$

$$V=\left(\begin{array}{cccc} a+d & 0 & 0 & 0\\ -ap & \gamma +d & 0 & 0\\ a\left(p-1\right) & 0 & d+\gamma_{SM}\left(1-p_{1}\right)+\lambda p_{1} & 0\\ 0 & 0 & -p_{1}\lambda & \gamma_{SS}+d \end{array}\right).$$

Since *J*_4_ is a lower triangular matrix, its eigenvalues are given by its diagonal entries, and due to the assumptions on our parameters, these real eigenvalues are all positive. Note that *J*_3_ does not play a role in the rest of the calculations, and therefore, we omit it. Finally, the inverse of *V* is given by the following equation:

$$V^{-1}=\left(\begin{array}{cccc} \frac{1}{a+d} & 0 & 0 & 0\\ \frac{ap}{\left(a+d\right)\left(\gamma +d\right)} & \frac{1}{\gamma +d} & 0 & 0\\ \frac{a\left(1-p\right)}{\left(a+d\right)\left(\gamma_{SM}+d-\gamma_{SM}p_{1}+\lambda p_{1}\right)} & 0 & \frac{1}{\gamma_{SM}+d-\gamma_{SM}p_{1}+\lambda p_{1}} & 0\\ \frac{a\lambda \left(1-p\right)p_{1}}{\left(a+d\right)\left(\gamma_{SS}+d\right)\left(\gamma_{SM}+d-\gamma_{SM}p_{1}+\lambda p_{1}\right)} & 0 & \frac{\lambda p_{1}}{\left(\gamma_{SM}+d\right)\left(\gamma_{SM}+d-\gamma_{SM}p_{1}+\lambda p_{1}\right)} & \frac{1}{\gamma_{SM}+d} \end{array}\right).$$

Clearly, *FV* ^−1^ is an upper triangular matrix with only one positive entry on its main diagonal. Therefore, **R**_0_, defined as the spectral radius, *ρ*(*FV* ^−1^), of *FV* ^−1^—the largest absolute value (or complex modulus) of its eigenvalues —, is given by the following equation:

(5) $$R_{0}=\underset{R_{0,I_{SM}}}{\underbrace{\frac{a\beta \Lambda (1-p)(d+\nu \sigma )}{d(a+d)(d+\nu )\left(\gamma_{SM}+d+p_{1}\left(\lambda -\gamma_{SM}\right)\right)}}}+\underset{R_{0,A}}{\underbrace{\frac{a\alpha \beta \Lambda p(d+\nu \sigma )}{d(a+d)(\gamma +d)(d+\nu )}}},$$

where all the parameters are defined in Table 2. From (5), it is clear that secondary infections are results of transmissions from mild symptomatic and asymptomatic individuals.

## 3 DISCUSSION

### 3.1 Application: Modelling the 2017–2018 diphtheria outbreak in Yemen

Diphtheria is a disease caused by toxin‐producing bacteria, *Corynebacterium diphtheriae*. The infection is spread from person to person through coughs or sneezes, or through close contact with an infected individual via respiratory droplets. The majority of infected individuals are asymptomatic or have a mild clinical course [18]. Symptoms usually begin 2–5 days after exposure, and they include fever, sore throat, swollen glands in the neck as well as difficulty with breathing and swallowing [14]. The toxin can cause a thick grey/white patch covering the throat [14]. In some cases, the disease can cause kidney failure, myocarditis, polyneuropathy and blockage to the airways [18]. Diphtheria can be treated via antibiotics that directly kill the bacteria or an antitoxin to stop the effects of the toxin. Furthermore, diphtheria is a vaccine preventable disease. However, it can be fatal even with treatment, and the case‐fatality rate in unvaccinated populations was estimated to be in the range of 5%–10% [19].

The Center for Disease Control and Prevention (CDC) recommends vaccination for all age groups to protect against diphtheria [20]. There are several types of vaccine depending on the age group of the individuals, for example, ‘DTaP’ for young children, ‘Tdap’ for adolescents and ‘Td’ or ‘Tdap’ for adults every 10 years as a booster vaccine. The World Health Organization emphasises the need for immunisation through vaccination for all children, globally, through a 3‐dose program [21], and reported that about 86% of children receive the required doses worldwide. With a full 3‐dose program, there is a vaccine efficacy of 97% according to the CDC [22].

### 3.2 The data

The data used in this study were collected in the Republic of Yemen, which is situated on the southernmost area of the Arabian Peninsula. Since 2014, the civil war in Yemen has had a severe impact on the country’s infrastructure, particularly in healthcare. There is a shortage of staff, equipment and medicines with approximately 50% of healthcare facilities fully functional in 2017 [23]. This is a key contributor to the declining vaccination coverage of the country, increasing the likelihood of being infected with vaccine‐preventable diseases such as diphtheria. In October 2017, a diphtheria outbreak was announced in the Republic of Yemen. The WHO reported, in February 2018, that 20 governorates had reported a total of 1085 cases and 66 deaths. About 30% of cases and 47% of deaths were recorded from children younger than the age of five [24]. In 2017, although the total population of the country, about 30 million people, was living in 22 governorates [25], the majority of diphtheria cases were reported in the governorates of Ibb and Al‐Hudayah with a combined population of around 1,139,000 [26, 27]. Hence, when we fit our model to data, we use *N* = 1,139,000 for the total population. Furthermore, a surveillance data analysis of the outbreak [12] for the entire country reports that, based on the vaccination status, the percentages of partially vaccinated, vaccinated, unvaccinated and unknown status patients were 6.6% (148/2243), 30.8% (690/2243), 48.6% (1090/2243) and 14.0% (315/2243), respectively. The zero dose reporting increased gradually by age from 35% in the age group <5 years to 74% in the age group >45 years, while three doses of the vaccine decreased gradually with age. Since the reported case fatality rate.

### 3.3 Parameter fitting

The number of weekly cases was extracted from ref. [28], and it is presented in Table 3.

**TABLE 3:** The number of diphtheria cases in Yemen over a 24‐week period in 2017–2018 [28].

Year	Week	# Cases
2017	Week 39	1
2017	Week 40	1
2017	Week 41	1
2017	Week 42	6
2017	Week 43	10
2017	Week 44	26
2017	Week 45	20
2017	Week 46	36
2017	Week 47	46
2017	Week 48	41
2017	Week 49	44
2017	Week 50	39
2017	Week 51	88
2017	Week 52	72
2018	Week 1	82
2018	Week 2	95
2018	Week 3	94
2018	Week 4	102
2018	Week 5	84
2018	Week 6	66
2018	Week 7	72
2018	Week 8	60
2018	Week 9	64
2018	Week 10	69

**FIGURE 2:**
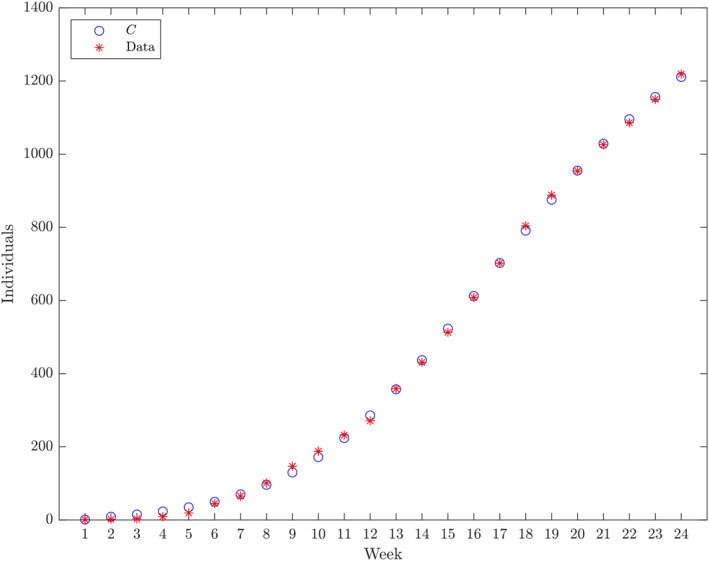
The cumulative number cases and the *C* component of the numerical solution of (6) using the initial condition $\left(S_{0},V_{0},E_{0},{I_{A}}_{0},{I_{SM}}_{0},{I_{SS}}_{0},R_{0},C_{0}\right)=\left(N-\tilde{V_{0}}-\tilde{E_{0}}-\tilde{{I_{A}}_{0}}-2-\tilde{R_{0}},\tilde{V_{0}},\tilde{E_{0}},\tilde{{I_{A}}_{0}},1,1,\tilde{R_{0}},1\right)$ and the parameters from Table 5. Horizontal axis: weeks from week 39 in 2017.

To estimate the initial conditions of the variables and the parameters listed in Tables 1 and 2, respectively, we fit the following extension of our model in (1)

(6) $$\begin{array}{cc} \overset{˙}{S} & =\Lambda N-\frac{\beta S\left(I_{SM}+\alpha I_{A}\right)}{N}-\nu S-dS\\ \overset{˙}{V} & =\nu S-\frac{\sigma \beta V\left(I_{SM}+\alpha I_{A}\right)}{N}-dV\\ \overset{˙}{E} & =\frac{\beta S\left(I_{SM}+\alpha I_{A}\right)}{N}+\frac{\sigma \beta V\left(I_{SM}+\alpha I_{A}\right)}{N}-aE-dE\\ {\overset{˙}{I}}_{A} & =paE-\gamma I_{A}-dI_{A}\\ {\overset{˙}{I}}_{SM} & =(1-p)aE-\left(1-p_{1}\right)\gamma_{SM}I_{SM}-p_{1}\lambda I_{SM}-dI_{SM}\\ {\overset{˙}{I}}_{SS} & =p_{1}\lambda I_{SM}-\gamma_{SS}I_{SS}-dI_{SS}\\ \overset{˙}{R} & =\gamma I_{A}+\left(1-p_{1}\right)\gamma_{SM}I_{SM}+\gamma_{SS}I_{SS}-dR\\ \overset{˙}{C} & =(1-p)aE, \end{array}$$

where the additional variable, *C*, captures the number of cumulative cases, and it is fitted to the number of cumulative cases obtained from the reported cases shown in Table 3. Notice that this variable does not affect the disease dynamics, and we use this component of the numerical solution of (6) to fit its parameters using the Matlab functions ode23 and lsqcurvefit [29]. Furthermore, since, in terms of the unknown functions in (6), we only have information about the size of the population and the number of positive cases, we also estimate the initial values of the components of (6) listed in Table 4, which provides the initial estimates together with the considered ranges. The initial estimates in Table 4 are generated randomly from the ranges provided in Tables 2 and 4. To initiate the fitting algorithm, the initial condition of (6) is set to,

$$\left(S_{0},V_{0},E_{0},{I_{A}}_{0},{I_{SM}}_{0},{I_{SS}}_{0},R_{0},C_{0}\right)=\left(N-V-E-I_{A}-2-R,V,E,I_{A},1,1,R,1\right),$$

where *V*, *E*, *I*_*A*_, *R* are the values in Table 4. By setting ${I_{SS}}_{0}=1$, we assume the presence of one unreported severe case when the first case as mild symptomatic is registered. Results of the parameter fitting process are summarised in Table 5. In Figure 2, we plotted the *C* component of the numerical solution of (6) together with the cumulative case numbers.

**TABLE 4:** Parameters, initial values of the compartments in (1), and their ranges used in Section 3.3.

	Initial estimate	Range
Parameter		
*β*	0.2	See Table 2
*ν*	$\frac{0.35}{365}$	See Table 2
*σ*	0.056	See Table 2
*α*	0.6	See Table 2
*a* ^−1^	3	See Table 2
*p*	0.6	See Table 2
*p*_1_	0.2	See Table 2
*λ* ^−1^	5	See Table 2
*γ* ^−1^	6	See Table 2
$\gamma_{SM}^{-1}$	5	See Table 2
$\gamma_{SS}^{-1}$	6	See Table 2
Compartment		
*V*	$\frac{3N}{4}$	$\frac{3N}{5}-\frac{9N}{10}$
*E*	10	1–20,000
*I*_*A*_	10	1–200
*R*	1.5 × 10^5^	$\frac{3N}{10}-\frac{81N}{100}$

**TABLE 5:** Fitted values of parameters and initial conditions of (6).

	Estimate	CI: 99.9%
Parameter		
*β*	0.8298	0.7261–0.9303
*ν*	0.0022	0.84514 × 10^−4^–0.0025
*σ*	0.0819	0.0707–0.1129
*α*	0.8114	0.7333–0.8492
*a*	0.3811	0.3051–0.4099
*p*	0.9938	0.9931–0.9968
*p*_1_	0.1196	0.1016–0.2786
*λ*	0.1397	0.0657–0.1439
*γ*	0.0588	0.0476–0.0592
*γ*_*SM*_	0.1390	0.0565–0.1882
*γ*_*SS*_	0.0935	0.0789–0.2036
**R**_**0**_	1.3267	1.2205–2.0317
Initial value		
$\tilde{V_{0}}$	6.8754 × 10^5^	6.7924 × 10^5^–7.0417 × 10^5^
$\tilde{E_{0}}$	919.324	807.2715–1946.7904
${\tilde{I_{A}}}_{0}$	199.8159	16.4157–239.7274
$\tilde{R_{0}}$	3.4302 × 10^5^	3.3867 × 10^5^–3.6696 × 10^5^

**FIGURE 3:**
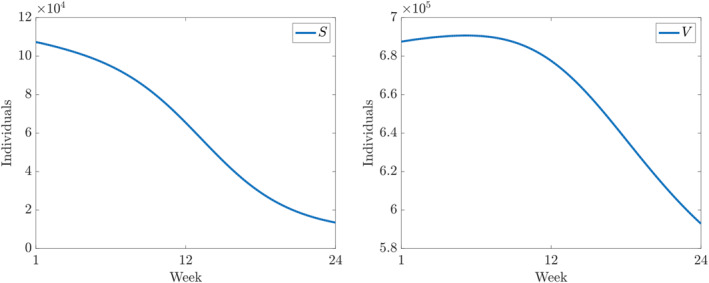
Numerical solutions of (6) using the fitted parameters in Table 5. Panel on the left and on the right shows the components *S* and *V*, respectively.

**FIGURE 4:**
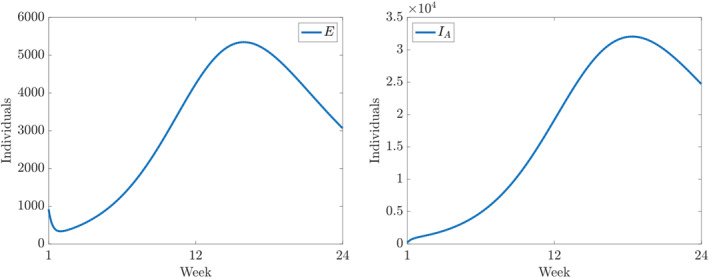
Numerical solutions of (6) using the fitted parameters in Table 5. Panels on the left and on the right show the components *E* and *I*_*A*_, respectively.

**FIGURE 5:**
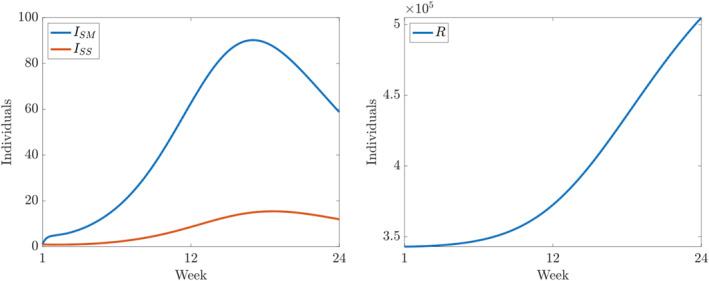
Numerical solutions of (6) using the fitted parameters in Table 5. Panels on the left and on the right show the components *I*_*SM*_ and *I*_*SS*_, and *R*, respectively.

Furthermore, the components of the numerical solution of (6) using the parameters and initial conditions in Table 5 are depicted in Figures 3, 4, 5.

Using the fitted parameters presented in Table 5 with (5), we obtain the following equation:

$$R_{0}\approx 1.3267.$$

**FIGURE 6:**
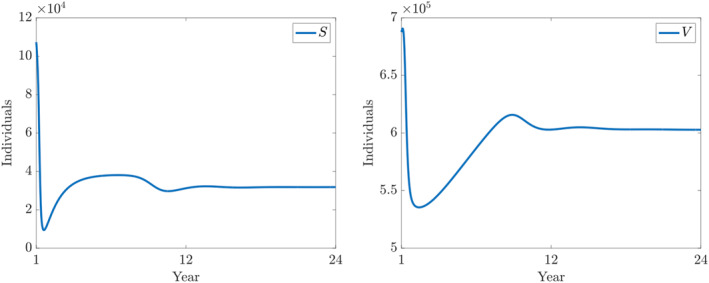
Numerical solutions of (6) using the fitted parameters in Table 5. Panels on the left and on the right show the components *S* and *V*, respectively.

**FIGURE 7:**
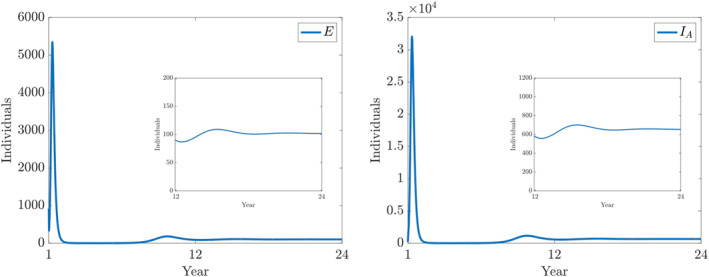
Numerical solutions of (6) using the fitted parameters in Table 5. Panels on the left and on the right show the components *E* and *I*_*A*_, respectively.

**FIGURE 8:**
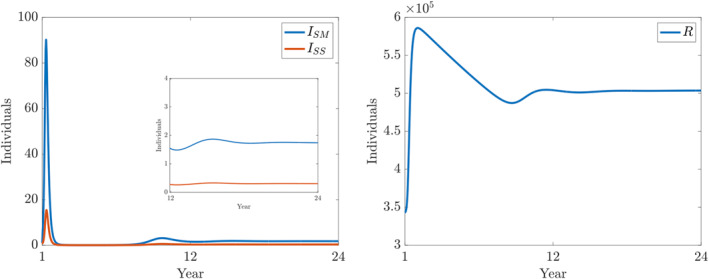
Numerical solutions of (6) using the fitted parameters in Table 5. Panels on the left and on the right show the components *I*_*SM*_ and *I*_*SS*_, and *R*, respectively.

It is important to note that (5) is valid only at the DFE. However, our results are based on a fitting process, which assumes non‐empty infected and removed compartments. In particular, on the left panel of Figure 4, the model suggests an initial decrease in *E*, the number of new infections. However, this is not in contradiction with **R**_0_ > 1 because of the results of **R**_0_ in ref. [11] are about the long‐term behaviour of solutions of differential equations used in mathematical modelling of infectious diseases. **R**_0_ > 1 implies the existence of at least one attractive object different from the DFE (which is unstable when **R**_0_ > 1). However, already the isolation of those objects can be a non‐trivial task which is outside of the scope of this paper, and we carry out this in a subsequent paper. Nevertheless, Figures 6, 7, 8 indicates that the solution of (6) with the fitted values in Table 5 converges to an endemic equilibrium.

**FIGURE 9:**
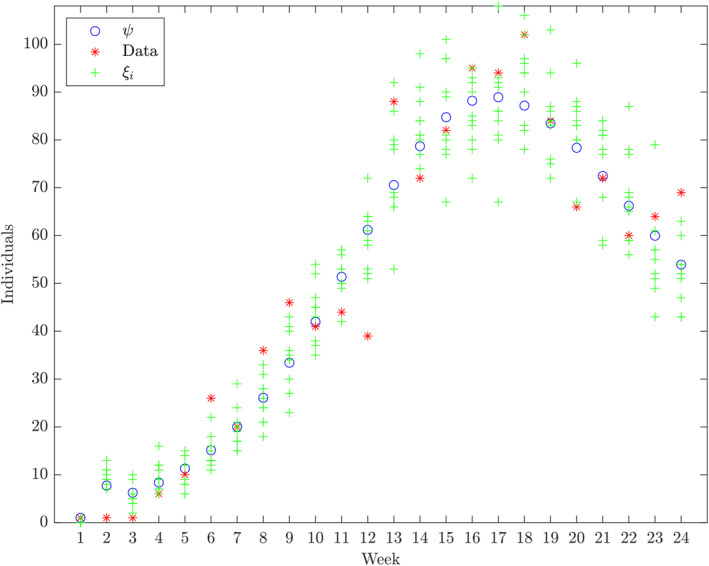
Illustration of the confidence interval generating bootstrap method with realisations of *ξ*_*i*_, *i* = 1, …, 10.

To address uncertainty both in data collection and modelling, based on ref. [30], we constructed 99.9% confidence intervals for the estimated values of the parameters and the initial values of the compartments in Table 5 as follows. From *C*(*t*), we derived the fitted weekly incidence of infection, $\psi ={\left\{\psi^{j}\right\}}_{j=1}^{24}$, and generated a new random sequence of daily incidence of infection, $\xi ={\left\{\xi^{j}\right\}}_{j=1}^{24}$ from the Poisson distribution specified by the rate parameter *ψ*_*j*_, *j* = 1, …, 24. Then, we fitted *C* of (5) to Ξ, the cumulative values of *ξ*, by applying the steps detailed above. We obtained candidate confidence intervals by using 10 realisations, *ξ*_*i*_, *i* = 1, …, 10, of *ξ* and the inverse cumulative distribution function of the Student’s *t* distribution. We repeated these steps until all the estimates in Table 5 were in the constructed candidate intervals. We provided a collection of such intervals in the last column of Table 5. The idea of the confidence interval candidate constructing algorithm is illustrated in Figure 9.

**FIGURE 10:**
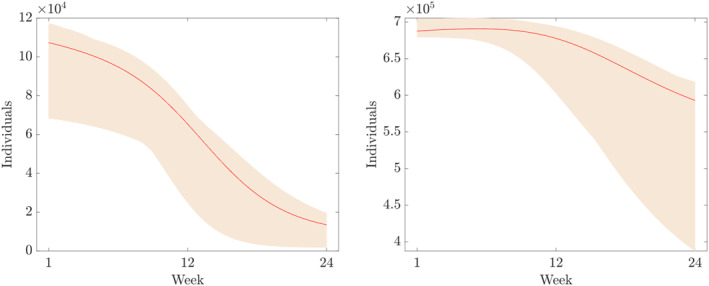
Numerical solutions of (6) using the fitted parameters in Table 5. Panel on the left and on the right shows the components *S* and *V*, respectively.

**FIGURE 11:**
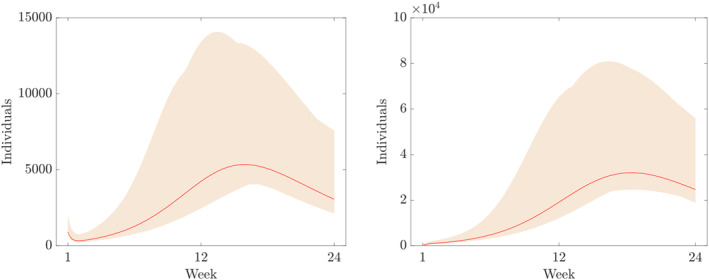
Numerical solutions of (6) using the fitted parameters in Table 5. Panel on the left and on the right shows the components *E* and *I*_*A*_, respectively.

**FIGURE 12:**
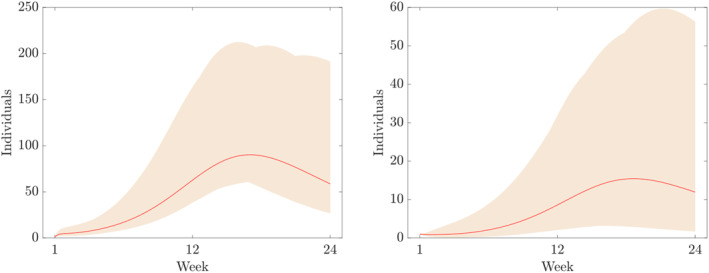
Numerical solutions of (6) using the fitted parameters in Table 5. Panel on the left and on the right shows the components *I*_*SM*_ and *I*_*SS*_, respectively.

**FIGURE 13:**
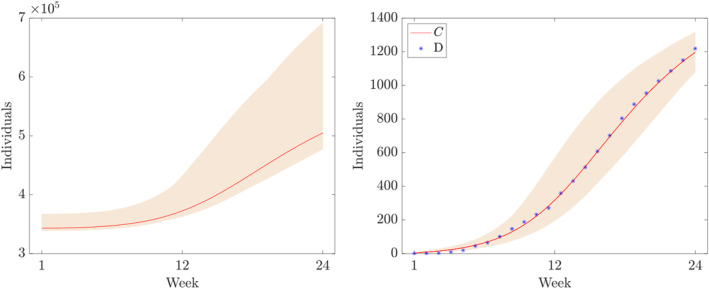
Numerical solutions of (6) using the fitted parameters in Table 5. Panel on the left and on the right shows the components *R* and variable *C*, respectively. The blue symbols mark the values from table.

To illustrate the impact of uncertainty both in data collection and modelling, using Latin Hypercube Sampling [31], we generated 5000 samples of parameters and initial conditions from the confidence intervals presented in Table 5 to generate numerical solutions of (6). From the set of solutions, we kept the 874 which satisfied the condition given below:

(7) $$D(24)\left(1-\frac{P}{100}\right)\leq C(24)\leq D(24)\left(1+\frac{P}{100}\right)$$

where *p* = 20 in this study, that is, we assume 20% overall uncertainty. Furthermore, *D* denotes the time series of the cumulative number of cases obtain from the data given in Table 3. In Figures 10, 11, 12, 13, we plot the coloured intervals between minimum and maximum for any *t* ∈  [1, 23] of these solutions, together with the solutions already presented in the figures above.

### 3.4 **R**_0_ sensitivity analysis

**FIGURE 14:**
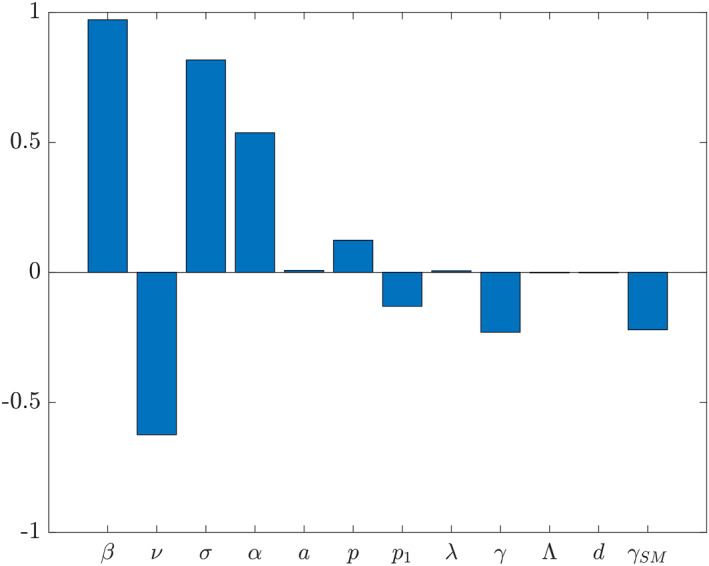
Partial rank correlation coefficients of the parameters with respect to **R**_0_ defined by (5).

Using Latin hypercube sampling, we generated a random set of 30,000 samples of the parameters in (5) from the intervals given in Table 2. Substituting these parameter values into (4), we obtained 30,000 values of **R**_0_.^4^ For the sensitivity analysis, we used the function pcc from the software R [32] to compute partial rank correlation coefficients to assess the effects of the parameters in Table 2 on **R**_0_. As shown in Figure 14, the transmission rate and the vaccination rate have largest absolute impacts on the basic reproduction number assuming that vaccine efficacy (1 − *σ*) cannot be increased further. Furthermore, while an increase in transmission rate increases **R**_0_, an increase in vaccination rate decreases **R**_0_. In addition, *σ* and *α* correlate positively with **R**_0_ since they represent transmission rate reduction in vaccinated and infectiousness reduction in asymptomatic individuals, respectively. That is, for the vaccinated individuals, because of its range in Table 2, and as it multiplies *β*, which can be seen as the base transmission rate in our model, a decreasing *σ* decreases the probability of the infection of a vaccinated individual. Therefore, 1 − *σ* can be seen as vaccine efficacy. Similarly, a decreasing *α* decreases the probability of that an asymptomatic person causes a new infection.

## 4 CONCLUSION

We derived and analysed a population‐based deterministic model for the spread of vaccine preventable infectious diseases featuring incubation period comparable with various infectious periods. The model is applicable to various communicable diseases when an asymptomatic disease spread is clinically evidenced, and symptomatic cases can progress from mild to severe cases. Another feature of the diseases to which our model is applicable is the lifelong natural immunity. We incorporated parameters to reflect the effects of imperfect vaccines, natural death, reduced transmission rates of vaccinated and asymptomatic cases, and we considered different recovery times from the relevant groups of individuals as well as the arrival of new susceptible subjects. In Section 2.1, we derived the basic reproduction number **R**_0_ for our model. In Section 3, we successfully fitted our model to the early phase of the 2017–2018 diphtheria outbreak in Yemen. Using our fitted parameters and the basic reproduction number of the model, we provided an estimate of **R**_0_. Furthermore, the estimation of the partial rank correlation coefficients of the parameters in (5) highlighted that a decrease of the transmission rate or an increase of the vaccination rate are the most effective ways of reducing **R**_0_ ^5^ provided that the most effective available vaccine is used.

The model can be extended to study diseases with more intricate transmission flows. For instance, when we applied the model in Section 3 to a diphtheria outbreak, we implicitly assumed that patients received a treatment appropriate to the stage of the disease progression. However, when more information becomes available about treatment protocols, one can incorporate various compartments to reflect the timings and the duration of the measures and controls in place to manage the infection. Also, we assumed that the incubation time of the symptomatic and asymptomatic cases is the same. Even if there is no information available about the latent period of the modelled disease, it can be incorporated using an additional parameter to represent different latent and incubation periods. Because of the relatively low level of the case fatality rate of diphtheria (5%–10% in an unvaccinated population) and the reported overall vaccination level in Yemen (which lowers the level of disease induced death even further), we did not consider disease induced death in this work. However, the case fatality rate can be significantly higher even in vaccine preventable diseases [33]. To model a disease with a high case‐fatality rate, such as the Influenza A virus subtype H5N1, the Ebola virus or the Marburg virus, one can introduce a parameter to represent the disease induced death rates at the relevant stages of the disease progression. In particular, when using our model (1), if we assume that disease induced deaths happen exclusively in the severe state, the number of severe cases, *I*_*SS*_, at any given time is governed by the following equation:

$${\dot{I}}_{SS}=p_{1}\lambda I_{SM}-\gamma_{SS}I_{SS}-\mu I_{ss}-dI_{SS},$$

where *μ* > 0 is the disease induced mortality rate. Also, we only considered two stages of the disease progression; however, this limitation can be resolved when an infection progresses through more clinically distinguishable stages by incorporating additional compartments reflecting a more detailed decomposition of the staged progression. This additional level of model accuracy, depending on the assumptions on the transmission routes, potentially increases the complexity of the basic reproduction number [34]. Finally, to consider the effects of the waning of the naturally acquired or vaccine provided immunity, one can extend the model by using an additional compartment comprising of individuals of reduced levels of protection, see, for example, ref. [35] or ref. [36].

The model can also serve as a basis of models, which consider different structures of the modelled population. For instance, when different age groups are impacted significantly and differently by a disease, an age structured model can capture the disease dynamics more faithfully. Similarly, age structured models can potentially provide an improved level of modelling accuracy if the timing of vaccination protocols are considered during the model building process. The age structure of a population can be considered, for example, using linked copies of the base model to represent a discrete age structure. The continuous age distribution of the population can be incorporated by coupling a partial differential equation to the differential equations describing the disease transmission, see, for example, refs. [37, 38]. Similarly, the spatial spread of a disease can be modelled by assuming that the considered population lives on possibly connected but separated patches when the so‐called meta‐population models are applicable, see ref. [39]. On the other hand, if the considered population is assumed to live without movement limiting barriers, partial differential equation based‐models can be used to study the spatial spread of infectious diseases [40].

## AUTHOR CONTRIBUTIONS

**Gabor Kiss**: Conceptualization; formal analysis; methodology; visualization; writing – review & editing. **Salissou Moutari**: Conceptualization; writing – review & editing. **Cara McTaggart**: Conceptualization; data curation; formal analysis; visualization; writing – original draft. **Lynsey Patterson**: Conceptualization; writing – review & editing. **Frank Kee**: Conceptualization; writing – review & editing. **Felicity Lamrock**: Conceptualization; writing – review & editing.

## CONFLICT OF INTEREST STATEMENT

The authors Gabor Kiss, Salissou Moutari, Cara McTaggart, Lynsey Patterson, Frank Kee and Felicity Lamrock declare that they have no conflicts of interest.

## ETHICS STATEMENT

This article does not contain any studies with human or animal subjects performed by any of the authors.

## ENDNOTES

^1^
Although [8] uses a more general approach, and the SIR model is only a special case therein, it is still very often referred to as the Kermack–McKendrick model, see also Ref. [41].

^2^
One can interpret 1 − *σ* as vaccine efficacy.

^3^
When fitting the model to real data, we assume that a new individual in *I*_*SM*_ is registered as a new case. That is, individuals, when entering *I*_*SS*_, are not registered as new cases.

^4^
The minimum and maximum of **R**_0_ of this sample are 0.0078 and 2.4842, respectively.

^5^
As the transmission rate naturally depends on contact rate, a reduction in contact rate implies a lower transmission rate.

## References

[1] Dietz K , Heesterbeek JAP . Daniel Bernoulli’s epidemiological model revisited. Math Biosci. 2002;180(1‐2):1–21. 12387913 10.1016/s0025-5564(02)00122-0

[2] Bernoulli D . Essai d’une nouvelle analyse de la mortalité causée par la petite vérole, et des avantages de l’inoculation pour la prévenir. Histoire de l’Acad., Roy. Sci. (Paris) avec Mem. 1760:1–45.

[3] Bacaër N , Bernoulli D . d’Alembert and the inoculation of smallpox (1760). London: Springer; 2011. p. 21–30.

[4] Brauer F , Van den Driessche P , Wu J , Allen LJS . Mathematical epidemiology, 1945. Springer; 2008.

[5] Murray JD . Mathematical biology: I. An introduction. Springer; 2002.

[6] Martcheva M . An introduction to mathematical epidemiology, 61. Springer; 2015.

[7] Viruses . Topical collection “mathematical modeling of viral infection”. 2020.

[8] Kermack WO , McKendrick AG . A contribution to the mathematical theory of epidemics. Proc R Soc Lond A Math Phys Sci. 1927;115(772):700–721.

[9] Hall PA , Kiss G , Kuhn T , Moutari S , Patterson E , Smith E . Mathematical modelling of the COVID‐19 epidemic in Northern Ireland in 2020. Open J Model Simulat. 2021;9(2):91–110.

[10] Hall PA , Kiss G , Kuhn T , Moutari S , Patterson E , Smith E . Estimating the level of asymptomatic COVID‐19 infections in Northern Ireland in 2020. Open J Model Simulat. 2022;10(2):190–218.

[11] Van den Driessche P , Watmough J . Reproduction numbers and sub‐threshold endemic equilibria for compartmental models of disease transmission. Math Biosci. 2002;180(1‐2):29–48. 12387915 10.1016/s0025-5564(02)00108-6

[12] Moghalles SA , Aboasba BA , Alamad MA , Khader YS . Epidemiology of diphtheria in Yemen, 2017–2018: surveillance data analysis. JMIR Public Health Surveill. 2021;7(6):e27590. 34076583 10.2196/27590 PMC8209531

[13] Truelove SA , Keegan LT , Moss WJ , Chaisson LH , Macher E , Azman AS , et al. Clinical and epidemiological aspects of diphtheria: a systematic review and pooled analysis. Clin Infect Dis. 2020;71(1):89–97. 31425581 10.1093/cid/ciz808 PMC7312233

[14] Diphtheria . Available at the website of NHS.

[15] Yemen . Yemen birth rate 1950–2023. Available at the website of Macrotrends.

[16] Hartman P . Ordinary differential equations. 2nd ed. Boston: Birkhäuser; 1982.

[17] Diekmann O , Heesterbeek JAP , Roberts MG . The construction of next‐generation matrices for compartmental epidemic models. J R Soc Interface. 2010;7(47):873–885. 19892718 10.1098/rsif.2009.0386 PMC2871801

[18] Diphtheria: causes and how it spreads. 2022. Available at the website of CDC in US.

[19] Clinical information. 2022. Available at the website of CDC in US.

[20] Diphtheria vaccination. 2022. Available at the website of CDC in US.

[21] Diphtheria . Available at the website of World Health Organization.

[22] About diphtheria, tetanus, and pertussis vaccination. 2022. Available at the website of CDC in US.

[23] Yemen: 2018 humanitarian needs overview ‐ Yemen. 2017. Available at the website of reliefweb.

[24] UN Office for the Coordination of Humanitarian Affairs . Yemen humanitarian update, covering 12–18 February 2018.

[25] United Nations, Department of Economic and Social Affairs, Population Division, Online Edition . World population prospects. 2022.

[26] Macrotrends LLC . Ibb, Yemen metro area population 1950–2023. 2023. Available at the website of Macrotrends.

[27] Macrotrends LLC . Al‐Hudaydah, Yemen metro area population 1950–2023. 2023. Available at the website of Macrotrends.

[28] Dureab F , Al‐Sakkaf M , Ismail O , Kuunibe N , Krisam J , Müller O , et al. Diphtheria outbreak in Yemen: the impact of conflict on a fragile health system. Conflict Health. 2019;13(1):1–7. 31139250 10.1186/s13031-019-0204-2 PMC6530011

[29] MATLAB . Version 9.13.0.2166757 (R2022b). Natick: The MathWorks Inc.; 2020.

[30] Efron B , Tibshirani R . Bootstrap methods for standard errors, confidence intervals, and other measures of statistical accuracy. Stat Sci. 1986:54–75.

[31] McKay MD , Beckman RJ , Conover WJ . A comparison of three methods for selecting values of input variables in the analysis of output from a computer code. Technometrics. 2000;42(1):55–61.

[32] Partial correlation coefficients. Available at the website of search. r‐project.

[33] Tiemersma EW , van der Werf MJ , Borgdorff MW , Williams BG , Nagelkerke NJD . Natural history of tuberculosis: duration and fatality of untreated pulmonary tuberculosis in HIV negative patients: a systematic review. PLoS One. 2011;6(4):e17601. 21483732 10.1371/journal.pone.0017601 PMC3070694

[34] Hyman JM , Jia L , Stanley EA . The differential infectivity and staged progression models for the transmission of HIV. Math Biosci. 1999;155(2):77–109. 10067074 10.1016/s0025-5564(98)10057-3

[35] Opoku‐Sarkodie R , Bartha FA , Polner M , Röst G . Dynamics of an SIRWS model with waning of immunity and varying immune boosting period. J Biol Dynam. 2022;16(1):596–618. 10.1080/17513758.2022.2109766 35943129

[36] Carlsson R‐M , Childs LM , Feng Z , Glasser JW , Heffernan JM , Li J , et al. Modeling the waning and boosting of immunity from infection or vaccination. J Theor Biol. 2020;497:110265. 32272134 10.1016/j.jtbi.2020.110265 PMC9108945

[37] Inaba H . Age‐structured population dynamics in demography and epidemiology. Springer; 2017.

[38] Cushing JM . An introduction to structured population dynamics. SIAM; 1998.

[39] Julien A . Spatio‐temporal spread of infectious pathogens of humans. Infect Dis Model. 2017;2(2):218–228. 29928738 10.1016/j.idm.2017.05.001 PMC6001966

[40] Rass L , Radcliffe J . Spatial deterministic epidemics. American Mathematical Soc.; 2003.

[41] Diekmann O , Heesterbeek JAP , Metz JAJ . The legacy of Kermack and McKendrick. Issue 5 of Publications of the Newton Institute, Volume 5 of Publications of the Newton Institute: Isaac Newton Institute for Mathematical Sciences. In: Mollison D , editor. Epidemic models: their structure and relation to data, 5. Cambridge University Press; 1995. p. 424.

